# WEE1 Inhibitors Mediate Antitumor Effects on Endometrial Cancer through Activation of Innate Immune Responses

**DOI:** 10.7150/jca.90236

**Published:** 2024-01-01

**Authors:** Yinuo Li, Xiangyu Wang, Xin Hou, Mingfu Wu, Shixuan Wang, Xiangyi Ma

**Affiliations:** Department of Obstetrics and Gynecology, Tongji Hospital, Tongji Medical College, Huazhong University of Science and Technology, Wuhan 430030, China.

**Keywords:** endometrial cancer, recurrence, target therapy, WEE1 inhibitor, innate immune response

## Abstract

**Introduction:** Recurrence signifies the primary mortality factor in patients suffering from endometrial cancer, with few efficacious treatments currently available for recurrent cases. This research investigates the anti-tumoral capacities of WEE1 inhibitors within the context of endometrial cancer, aiming to establish a novel therapeutic avenue for high recurrence-risk patients.

**Materials and methods:** We evaluated WEE1 expression in endometrial cancer patients utilizing immunohistochemistry on paraffin-embedded tissue sections. The cytotoxic potential of WEE1 inhibitors on endometrial cancer cells was assessed by CCK8 assay. Assays to gauge the influence of WEE1 inhibitors on cell proliferation and migration included clonal proliferation, wound healing, and transwell assays. We determined the impacts on apoptosis and cell cycle stages by flow cytometry. Employing qRT-PCR and western blotting, we investigated the mechanistic pathways underlying the anti-tumoral activity of WEE1 inhibitors. *In vivo* evaluations were executed to ascertain the inhibitory effect of WEE1 inhibitors on tumor growth in mice.

**Results:** WEE1 exhibited high-level expression in endometrial cancer tissues, particularly pronounced in recurrent compared with non-recurrent patients. WEE1 inhibitors effectively eliminated endometrial cancer cells while inhibiting their proliferation and migration. Flow cytometric analyses revealed a significant promotion of apoptosis and an increase in G2/M phase cell proportion upon WEE1 inhibitor treatment. qRT-PCR and western blotting elucidated that WEE1 inhibitors activated the innate immune signaling pathway in endometrial cancer cells. Furthermore, *in vivo* assessments demonstrated substantial tumor growth suppression due to WEE1 inhibitors.

**Conclusions:** WEE1 inhibitors initiated an innate immune response in endometrial cancer, exhibiting considerable anti-tumoral effects, which was promising for postoperative treatment of endometrial cancer, especially recurrent endometrial cancer patients.

## Introduction

Endometrial cancer (EC) is one of the three major gynecologic malignant tumors and the sixth most common cancer in female. In 2020, there were 417,367 new endometrial cancer cases worldwide, culminating in 97,370 fatalities^1^. Particularly in the United States, endometrial cancer tops the chart amongst female reproductive system malignancies, with an estimated 66,200 new cases and 13,030 deaths projected for 2023^2^. In China, both the incidence and mortality rates of endometrial cancer continue to escalate annually. Approximately 70% of endometrial cancer patients present with tumors localized within the uterine body and are diagnosed at an early stage, thus procuring favorable clinical outcomes following timely surgical intervention^3^, ^4^, with 5-year overall survival ranging from 74% to 91%^5^. However, roughly 20% of endometrial cancer patients experience disease recurrence post initial treatment, with 75% of such instances occurring within the initial 2-3 years following diagnosis. Advanced endometrial cancers tend to exhibit a more malignant phenotype, leading to a staggering 5-year recurrence rate of 59%^6^. Patients with recurrent tumors face bleak prognoses, with a severe dip in long-term survival rates, the leading cause of death from endometrial cancer^7^.

Over the past several decades, the diagnostic methodologies and therapeutic approaches for endometrial cancer have progressively improved. Surgery remains the preferred treatment modality^8^, with adjunct vaginal brachytherapy or pelvic radiation therapy proved effective in reducing local recurrence and improving progression-free survival. Chemotherapy serves as a systemic adjuvant therapy, particularly suitable for patients with advanced, recurrent, or metastatic endometrial cancer, as well as those at high risk of postoperative recurrence. The implementation of chemotherapy, however, remains a subject of controversy^9^. As unopposed estrogen stimulation is closely tied to endometrial cancer development, progesterone therapy holds substantial merit in postoperative adjuvant therapy^10^. Nevertheless, once endometrial cancer recurs, traditional treatments such as surgery, radiotherapy, chemotherapy, and hormonal therapies fail to extend patient survival, necessitating the urgent exploration of more effective therapeutic strategies to enhance the life quality of patients with recurrent endometrial cancer.

Recently, targeted therapy has seen rapid expansion, demonstrating substantial anti-tumor activity in various cancers and preliminary efficacy in endometrial cancer^11^. In 2022, an article published in* Lancet* highlighted targeted therapy as a burgeoning area of focus within the realm of endometrial cancer treatment. In the upcoming years, a multitude of clinical and preclinical studies will investigate the clinical benefits of targeted therapy in advanced, recurrent, and metastatic endometrial cancer^12^. WEE1 inhibitors, emerging as a promising targeted therapeutic approach, have been the focus of extensive attention in recent years. WEE1 kinase, the critical regulator of the G2/M cell cycle checkpoint and replication stress response^13^, chiefly modulates cell cycle protein-dependent kinase 1, thus facilitating the re-entry of the cell cycle after DNA repair, ultimately ensuring proper cell cycle progression^14^. Correspondingly, inhibiting WEE1 kinase activity leads to a significant amount of unrepaired damaged DNA entering the cell cycle, triggering mitotic catastrophe, and subsequently, tumor cell apoptosis. The first phase I clinical trial of a WEE1 inhibitor was conducted in 2015, targeting multiple refractory solid tumors^15^. Among gynecological cancers, its efficacy and safety as a new targeted drug have been investigated extensively in ovarian cancer through various basic studies and clinical trials^16^-^19^. However, studies on WEE1 inhibitors in endometrial cancer remain in nascent stages. An initial phase II clinical trial evaluating the anti-tumor effects of a WEE1 inhibitor in recurrent serous endometrial carcinoma found that recurrence-free survival at 6 months achieved 47.1%, suggesting the potential therapeutic promise of WEE1 inhibitors in endometrial cancer^20^.

Hence, in this study, we aim to delve into the anti-tumor effects of WEE1 inhibitors and associated mechanisms in endometrial cancer comprehensively, in hopes of offering a viable treatment for patients with endometrial cancer, particularly those prone to relapse and metastasis.

## Material and Methods

### Cell lines and culture

This study employed five human-derived endometrial cancer cell lines: HEC-1-A, HEC-1-B, ISHIKAWA, RL95-2, and KLE. HEC-1-A and ISHIKAWA were sourced from the Cell Bank of the Chinese Academy of Sciences (China) and Wuhan Procell Science and Technology Co., Ltd. (China) respectively. HEC-1-B and RL95-2 were purchased from American Tissue Culture Collection (ATCC, USA), and KLE was purchased from the China Center for Type Culture Collection (China). The HEC-1-A was cultured in McCoy's 5A medium containing 10% fetal bovine serum. Both HEC-1-B and ISHIKAWA were cultured in DMEM medium containing 10% fetal bovine serum, whereas RL95-2 and KLE were cultured in DMEM/F12 medium, also supplemented with 10% fetal bovine serum. All cell lines, being adherent growth cells, were cultured at 37°C in a cell culture incubator with 5% CO2 and 90% relative humidity.

### CCK8 assay

Post digestion into single-cell suspension, cells were counted and seeded in 96-well plates at a concentration of 5-7×10^3^/100μl per well. Once adhered, cells were treated with either DMSO (control) or AZD1775 (experimental). AZD1775, initially dissolved in DMSO, was diluted in a 1:3 ratio, setting up more than three replicates for each concentration. Post 72-hour drug treatment, cells were exposed to a CCK8 solution (CCK8: medium=1:9) and incubated in a light-protected cell culture incubator. In parallel, four cell-free wells were set as blank control. Absorbance at 450 nm (OD value) was measured using a multifunctional microplate reader at 1 h, 2 h, and 3 h following the addition of the CCK8 solution. Using untreated cells as a control (set survival rate at 100%), the survival rate of drug-treated cells was calculated relative to the control. The obtained values were then used to plot a cell inhibition rate curve in GraphPad Prism 8 and calculate the IC50 of AZD1775 in different cells.

### Clonal proliferation assay

After counting the single-cell suspension obtained from cell digestion, cells were seeded in 12-well plates at a concentration of 1,000 cells (range 800-1500) per well in 1.5 mL medium. Cells, post adhesion, were treated with either DMSO (control) or AZD1775 (experimental). Following a 5-day incubation, the medium was replaced and incubation continued until cells reached an appropriate density. After washing twice with PBS, cells were fixed using paraformaldehyde for a minimum of 15 minutes at room temperature. Fixed cells were washed again with PBS and stained with crystal violet dye. After drying at room temperature, plates were scanned and images captured using an Epson scanner. (Japan).

### Wound healing assay

Cells, after being digested into single-cell suspension, were plated in 6-well plates. At approximately 90% cell density, a straight wound was created using a sterile 100ul pipette tip under a ruler. Free cells, dead cells, and cell debris from the wound area were gently rinsed away with sterile PBS. Post adding medium, plates were placed in an incubator. The healing of the wound was visualized using an electron microscope. Images of the wounds were captured at 0 h, 24 h, and 48 h post wound creation, and analyzed using ImageJ.

### Transwell assay

Cells were digested, centrifuged, and resuspended in serum-free medium, then counted under a microscope. Cells were seeded in Transwell chambers at a concentration of 8×10^4^ cells per 200μl per well. The lower section of the 24-well plate was filled with 700μl medium supplemented with 20% serum, upon which the chamber containing cell suspension was placed. After 24 h and 48 h of incubation at 37°C, cells were stained following two washes with PBS. Formaldehyde was used to fix cells. Non-migrated cells on the upper surface of the chamber were removed using a cotton swab. Chambers were stained with crystal violet in the dark at room temperature for 30 minutes. Post drying, chambers were mounted on glass slides and scanned using a slide scanner (EPSON, Japan).

### Apoptosis Detection by Flow Cytometry

Cells were seeded in 6-well plates and treated with either DMSO (control group) or AZD1775 (experimental group) after cell adhesion. The cells were incubated in medium for 72 hours post drug treatment. The supernatant was then collected, and cells in the plate were digested with pancreatin. The digested cells were combined with the supernatant and centrifuged. Cells were washed with PBS, and the supernatant was discarded. Binding buffer (1×) was prepared by diluting 10× binding buffer with PBS buffer, which was then used to resuspend the cell pellet in each tube. Groups were set up as follows: no dye was added to the blank group, 5μL AnnexinV-FITC was added to the FITC single standard group, 10μL Propidium Iodide stain was added to the Propidium Iodide single standard group, and both 5μL AnnexinV-FITC and 10μL Propidium Iodide dye were added in the other groups. These were then incubated for 20 min at room temperature, protected from light. Apoptosis was measured using flow cytometry, and results were analyzed using FlowJo 10.

### Cell Cycle Detection via Flow Cytometry

Cells were seeded in 6-well plates and treated with corresponding drugs post cell adhesion. After 72 hours of continued culture in the medium post-treatment, the cell supernatant was discarded. The adhered cells were enzymatically dissociated with pancreatin, a process that was halted by the addition of the corresponding medium. The cell suspension obtained from digestion was transferred to a centrifuge tube for centrifugation. The collected cell pellet was rinsed with PBS, discarding the supernatant. The pellet was then resuspended in 120μL of PBS per tube. This cell suspension was slowly added to pre-cooled 75% ethanol and incubated at 4℃ overnight. The cell suspension was rewarmed to room temperature, centrifuged, and the supernatant discarded. The cell pellet was rinsed with PBS, and a 1% BSA solution prepared in PBS was used to create a membrane-permeabilizing solution containing 0.25% Triton. Each sample received 200-300μL of this solution, and post-centrifugation, the supernatant was discarded. The remaining cell mass was treated with 1% BSA solution, and post-centrifugation, the supernatant was discarded. Each tube was supplemented with 200-300 Propidium Iodide /RNase Staining Buffer according to the number of cells in each tube. Following a 20-minute incubation at room temperature protected from light, the number of cells at different time points was measured by flow cytometry, and the results were analyzed using FlowJo 10.

### Western blotting

The collected cell sediment was resuspended with an appropriate amount of RIPA lysis solution spiked with protease inhibitors and lysed with an ultrasonic instrument. The protein concentrations of extracts were measured by the Bradford method after centrifugation. Protein was separated by sodium dodecyl sulfate-polyacrylamide gel electrophoresis and then transferred to polyvinylidene difluoride membranes (Merck Millipore, USA). The polyvinylidene difluoride membranes were blocked with 5% bovine serum albumin for 1 h at room temperature and incubated with primary antibodies overnight at 4°C in a refrigerator. The next day, membranes were incubated with secondary antibody for 1 h at room temperature. Electrochemiluminescence (Bio-Rad, USA) and detection reagents (Advansta, China) were used to detect signals. The antibodies used for western blotting were listed in Table S1.

### Quantitative RT-PCR (qRT-PCR)

Total RNA was extracted using the RNA Extraction Kit (Vazyme Biotech Co., Ltd, China), following the manufacturer's instructions. The RNA concentration of each sample was measured, and 1000ng of RNA was reverse-transcribed to cDNA. qRT-PCR was performed on a quantitative real-time PCR System (Bio-Rad, Amercia) using the ChamQ Universal SYBR qPCR Master Mix (Vazyme Biotech Co.,Ltd, China). Gene expression level were normalized to β-actin. The primer sequences used in this study were provided in Table S2.

### Immunohistochemistry

Tumor samples from endometrial cancer patients were fixed in 4% paraformaldehyde overnight, embedded in paraffin, and sectioned. The sections underwent baking in a 65℃ oven for over an hour, followed by xylene dewaxing and ethanol-based rehydration. After triple washing with PBS, high-temperature antigen retrieval was performed using EDTA solution. Then they were incubated with 3% H_2_O_2_ to block endogenous peroxidase after cooling. Next, sections were treated with ready-to-use goat serum at 37℃. As per antibody instructions, the primary antibody (antiWEE1, Ab288727, Abcam) was diluted appropriately in PBS. Each tissue section was incubated with 50-100 μL of this solution in a humidified chamber at 4℃ overnight. After reaching room temperature, sections were treated with biotin-labeled secondary antibodies at 37°C. Horseradish peroxidase-labeled streptavidin working solution was added to the sections, followed by a 15-minute incubation at room temperature, and DAB chromogen-based coloration. The sections were then counterstained with hematoxylin, differentiated in 1% hydrochloric acid alcohol, and blued in ammonia. Following sequential immersion in graded ethanol and xylene for dehydration and clarification, the sections were air-dried, mounted, and scanned using a slide scanner (EPSON, Japan).

### Animal experiment

Endometrial cancer cells were enzymatically dissociated to a single-cell suspension and injected subcutaneously into healthy NCG mice at a concentration of 1×10^7^ cells per mouse. Regular observations were made to monitor the health status of the mice and tumor development. Once the tumors reached approximately 2mm in size, the mice were randomly assigned to two groups for drug administration. The vehicle solution consisted of 2% DMSO, 30% PEG300, 5% TWEEN80, and sterile PBS. The control group received only the vehicle solution while the treated group was administered AZD1775 at a dose of 60 mg/kg/d for five consecutive days, followed by a two-day break. Adherence to this regimen enabled the monitoring of mice body weight and subcutaneous tumor size every three days over a span of 28 days. At the conclusion of the study, mice were euthanized. The variations in body weight and tumor volume were plotted over time.

### Statistics analysis

All experiments were independently performed at least three times. Data were analyzed statistically using GraphPad Prism 8 software, and values are presented as the mean ± standard deviation. The t-test was employed to assess the differences between two datasets, and one-way ANOVA was used for the comparison of multiple datasets. The statistical significance was determined when P<0.05. In this regard, *, **, ***, ****, and ns represent P<0.05, P<0.01, P<0.001, P<0.0001, and not statistically significant, respectively.

### Ethical approval

This study was approved by the Institutional Review Committee of Tongji Hospital, Tongji Medical College, Huazhong University of Science and Technology (No. TJ-IRB20220556). The study was performed in compliance with the Declaration of Helsinki. Written informed consent was obtained from the patients to participate in this study prior to the investigation.

## Results

### WEE1 Overexpression in Tissues from Endometrial Cancer Patients, Especially Those with Recurrent Diseases

**Figure 1:**
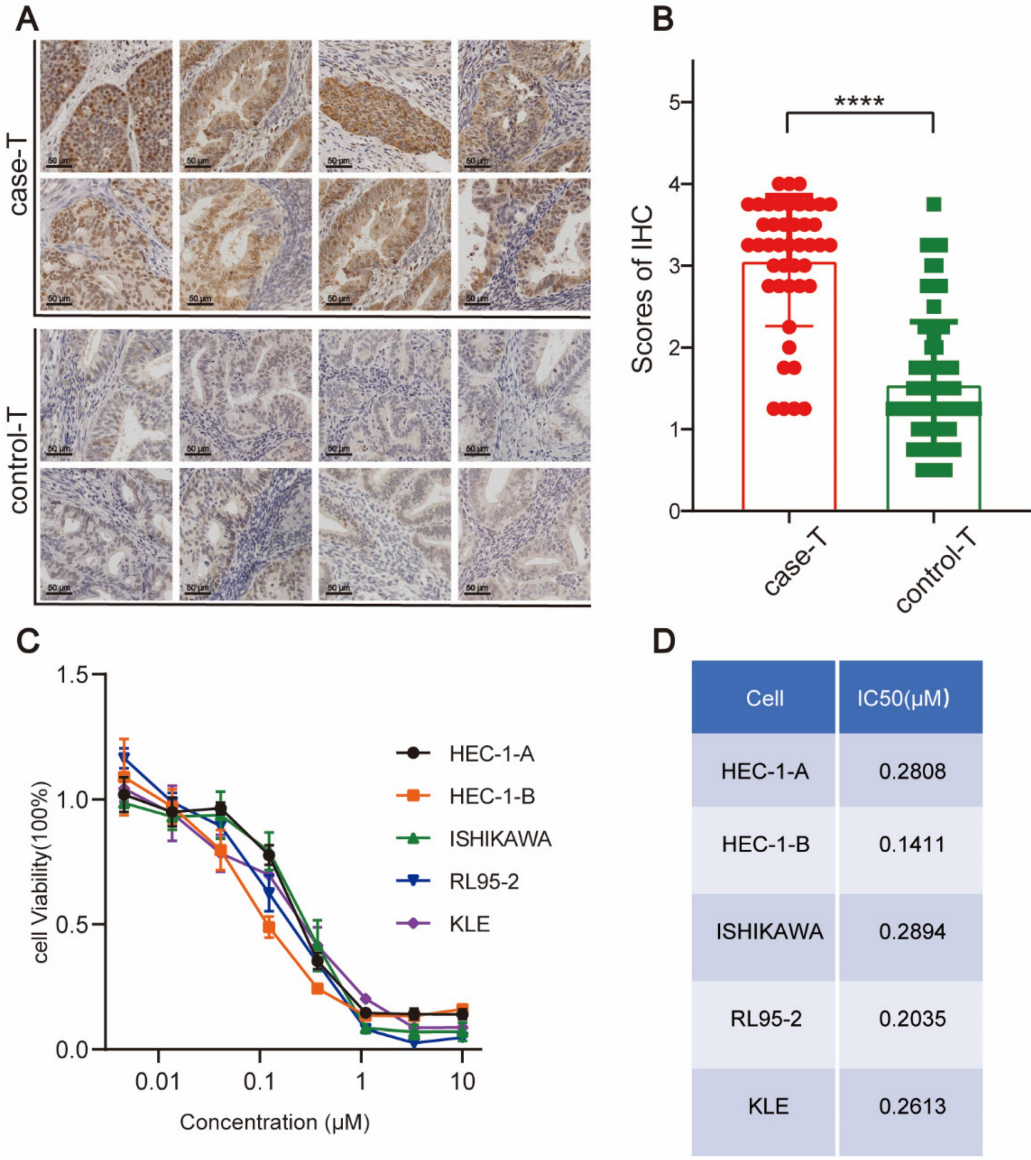
WEE1 Protein Expression in Endometrial Cancer and the Killing Effect of AZD1775 in Endometrial Cancer. A: Immunohistochemical staining showcased WEE1 protein expression in cancer foci of patients with recurrent (top) versus non-recurrent (bottom) endometrial cancer. B: Quantitative assessment of WEE1 protein staining in cancer foci of recurrent and non-recurrent patients. Data were presented as mean ± standard deviation, analyzed via t-test. **** denoted P<0.0001. C: Endometrial cancer cell lines (HEC-1-A, HEC-1-B, ISHIKAWA, RL95-2, KLE) were cultured in 96-well plates, treated with either DMSO (control) or varying concentrations of AZD1775 (max 10 μM, decreasing in 3-fold increments across 8 gradients). Cell viability was assessed with the CCK8 kit post 72 hours. D: The half-maximal inhibitory concentrations (IC50) of AZD1775 across the cell lines were determined using GraphPad 8.

WEE1 regulates the cell cycle progression at the G2/M phases, providing cells ample time to repair damaged DNA before proceeding to the next division cycle. Accumulating evidence indicates that aberrant WEE1 expression holds prognostic value in various cancers. Slipicevic et al. 21 utilized immunohistochemistry to assess WEE1 expression in ovarian cancer, revealing that recurrent patients, particularly those with prior chemotherapy experience, exhibited elevated WEE1 levels, correlating with reduced overall survival. Given that tumors in recurrent endometrial cancer patients tend to display greater malignancy and likely require increased WEE1 for DNA repair, we investigated WEE1 expression in tumor tissues from recurrent and non-recurrent endometrial cancer patients. The results suggested WEE1 was expressed in all tumor tissues of endometrial cancer patients (Figure 1A). Notably, the expression level of WEE1 in tumor tissues of recurrent patients was relatively higher than that of non-recurrent patients (Figure 1B), which illustrated in endometrial cancer, the expression of WEE1 was prognostically important and the tumor in recurrent patients might require more WEE1 to repair the large amount of damaged DNA in replication process, to maintain the tumor cell proliferation. We aimed to examine if WEE1 inhibition could elicit anti-tumor effects and its underlying mechanisms, with the hope of introducing a novel postoperative treatment strategy for endometrial cancer patients, particularly those experiencing recurrence.

### WEE1 Inhibitors Exert Cytotoxic Effects on Endometrial Cancer Cell Lines

AZD1775, the WEE1 inhibitor used in this study, has shown promise in preclinical and clinical trials due to its ability to abrogate G2/M checkpoints, leading to mitotic catastrophe by allowing cells with damaged DNA to enter mitosis. We selected five human-derived endometrial cancer cell lines, namely HEC-1-A, HEC-1-B, ISHIKAWA, RL95-2, and KLE, for our experiments.

We conducted a CCK8 assay to evaluate the cytotoxic effect of AZD1775 on these cell lines, utilizing a maximum concentration of 10 μM, with serial dilutions at a 1:3 ratio across eight distinct concentrations. Our results indicated a dose-dependent decrease in cell survival, consistent across all cell lines, thereby affirming AZD1775's robust cytotoxic effect on the five endometrial cancer cell lines (Figure 1C). Moreover, the IC50 of AZD1775 in all these cell lines was in the nanomolar range, indicating that endometrial cancer cells are susceptible to AZD1775 (Figure 1D).

### WEE1 Inhibitors Impede Proliferation and Migration of Endometrial Cancer Cells

**Figure 2:**
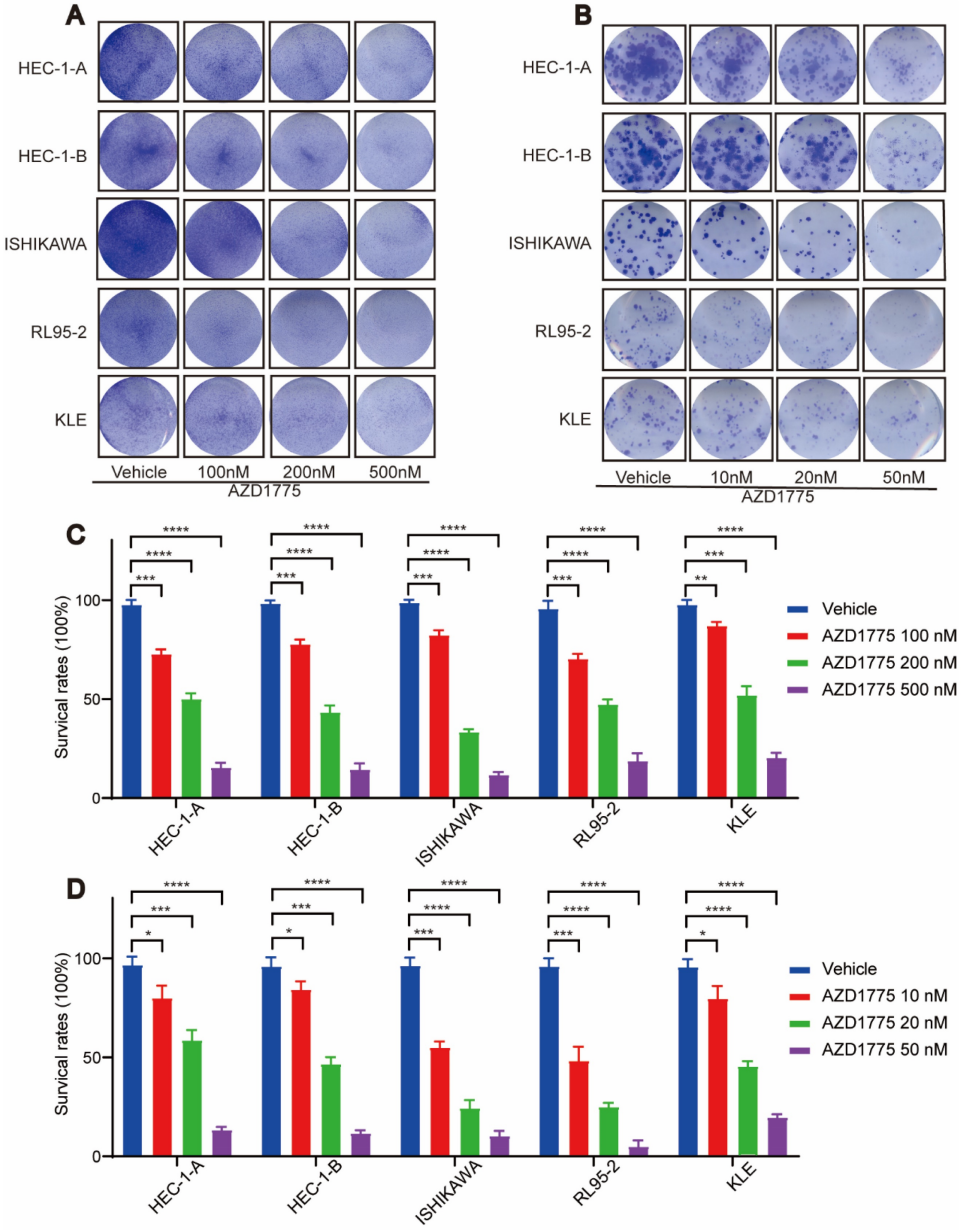
Effect of AZD1775 on Proliferation of Endometrial Cancer Cells. A: Endometrial cancer cell lines (HEC-1-A, HEC-1-B, ISHIKAWA, RL95-2, KLE) were cultured in 12-well plates and treated with DMSO or AZD1775 at concentrations of 100 nM, 200 nM, or 500 nM. Post 72 hours, cells were stained with crystal violet and imaged. B: The aforementioned cell lines were seeded in 12-well plates at densities of 800, 1,000, 800, 1,200, and 1,500 cells, respectively. They were then treated with DMSO or AZD1775 at concentrations of 10 nM, 20 nM, or 50 nM and stained with crystal violet. Imaging was conducted after 13, 13, 13, 17, and 18 days correspondingly. C & D: Using the DMSO-treated group as the control (100% cell survival), the survival rates of cells treated with various AZD1775 concentrations were evaluated. Data were presented as mean ± standard deviation, analyzed via t-test. Significance levels were indicated as * (P<0.05), ** (P<0.01), *** (P<0.001), and **** (P<0.0001).

**Figure 3:**
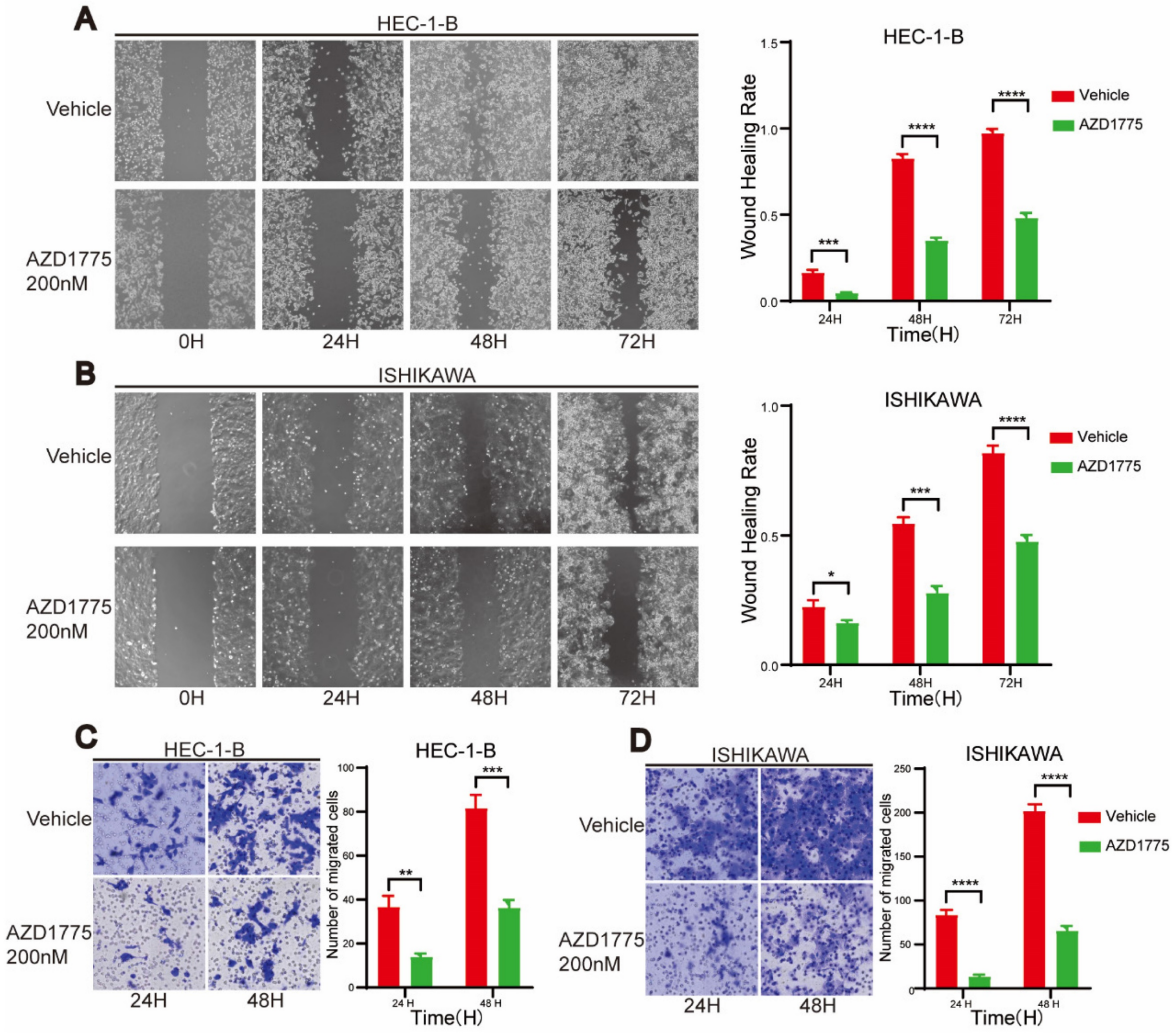
Impact of AZD1775 on Migration of Endometrial Cancer Cells. A & B: HEC-1-B and ISHIKAWA were cultured in 6-well plates, and a straight wound was drawn when the cell density reached 90%, and DMSO and 200nM AZD1775 were given respectively. Cells in different treatment groups were photographed at 24H, 48H and 72H, respectively. On the right side, the statistical analysis of cell healing in the two groups at different time periods was shown. C & D: HEC-1-B and ISHIKAWA were cultured in transwell chambers and given DMSO and 200nM of AZD1775 treatment, respectively. Cells from different treatment groups were stained with crystal violet and scanned after 24H and 48H, respectively. Statistical analysis of cell migration in the two groups at different time periods was shown on the right. The experimental data were expressed as mean ± standard deviation and statistically analyzed by t-test, * represented P<0.05, ** represented P<0.01, *** represented P<0.001, and **** represented P<0.0001.

In examining the impact of WEE1 inhibitors on endometrial cancer progression, we conducted clone formation, wound healing, and transwell assays. The clone formation assay revealed that, in comparison to the control group, short-term AZD1775 treatment at high concentrations of 100nM, 200nM, and 500nM suppressed the proliferation of endometrial cell lines in the experimental group (Figure 2A, Figure 2C). Furthermore, long-term exposure to low concentrations of 10nM, 20nM, and 50nM of AZD1775 inhibited cell proliferation in a concentration-dependent manner (Figure 2B, Figure 2D). The wound healing assay corroborated these findings, illustrating that WEE1 inhibitors significantly curtailed endometrial cell proliferation (Figure 3A), with the inhibitory effects intensifying over time (Figure 3B). Considering that cancer cell migration is a crucial factor in tumor progression, HEC-1-B and ISHIKAWA cells were treated with 200nM of AZD1775. The results indicated that WEE1 inhibitors hindered the migration of endometrial cancer cells (Figure 3C, Figure 3D). Overall, these findings suggest that AZD1775 can inhibit the proliferation and migration of endometrial cancer cells, thereby delaying disease progression.

### WEE1 Inhibitors Induce Apoptosis in Endometrial Cancer Cells

As WEE1 kinase plays a significant role in DNA damage repair, its inhibition can increase genomic instability and eventually induce apoptosis. Consequently, this study also explored whether AZD1775 could promote apoptosis and contribute to tumor suppression. Five endometrial cancer cell lines were treated with various concentrations of AZD1775, namely 100nM, 200nM, 500nM and 1μM. We conducted flow cytometry assay to determine the effect of AZD1775 on cell apoptosis and performed western blotting before and after drug administration to evaluate changes in the apoptotic marker caspase 3.

**Figure 4:**
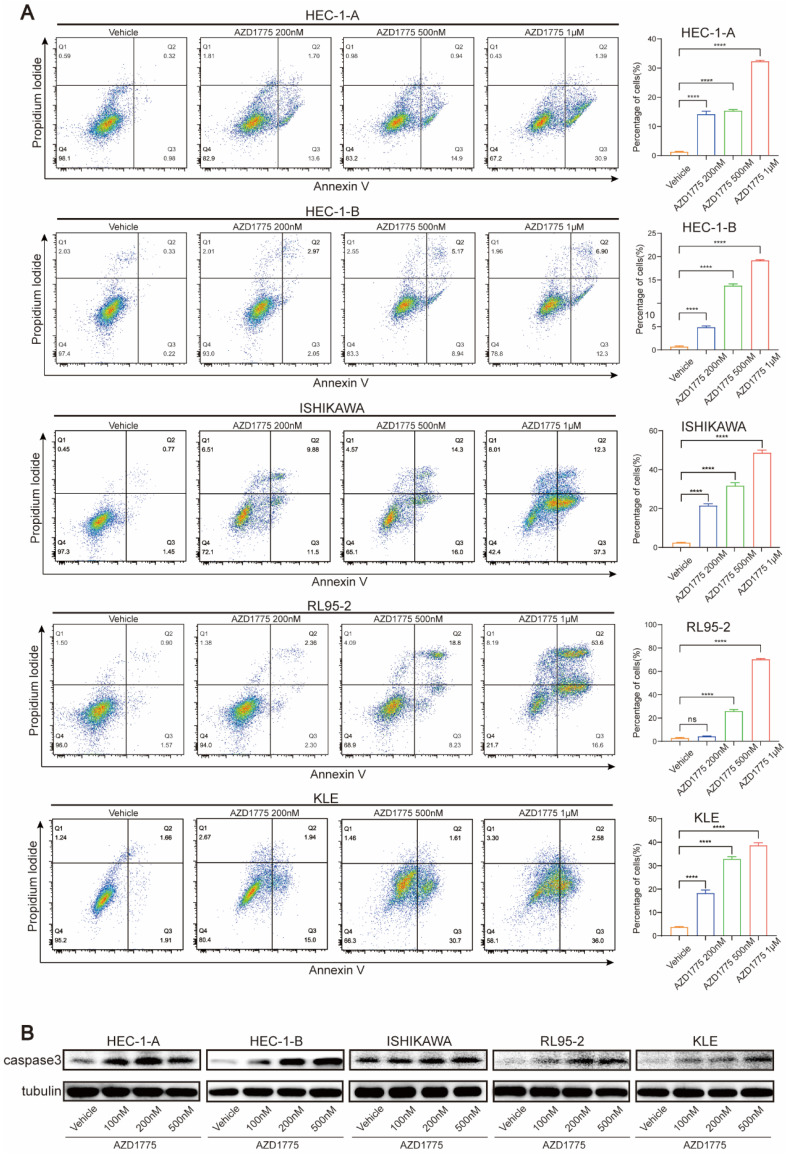
Influence of AZD1775 on Apoptosis in Endometrial Cancer Cells. A: Endometrial cancer cell lines HEC-1-A, HEC-1-B, ISHIKAWA, RL95-2, and KLE were seeded in 6-well plates and treated with DMSO, 200 nM, 500 nM, and 1 μM of AZD1775. Post 72h, apoptosis was assessed using flow cytometry. B: The aforementioned cell lines were exposed to varying concentrations of AZD1775 for 72h, after which changes in caspase 3 protein levels were determined using a Western Blotting assay. Data were presented as mean ± standard deviation and analyzed using a t-test. Notations: **** denoted P<0.0001; ns indicated no statistical significance.

Flow cytometry analysis revealed that 100nM AZD1775 did not significantly induce apoptosis across the cell lines (data not shown), prompting an increase in drug concentration for subsequent experiments. Figure 4A demonstrates that AZD1775 induced significant apoptosis from 200nM onwards in all cell lines except for RL95-2, with the effects intensifying as the drug concentration increased (Figure 4B). Western blotting analysis showed a concentration-dependent upregulation of caspase 3 expression following AZD1775 treatment (Figure 4C). These findings indicate that AZD1775 can trigger apoptosis in endometrial cancer cells, with the pro-apoptotic effect correlating positively with drug concentration.

### WEE1 Inhibitors Induce G2/M Cell Cycle Arrest in Endometrial Cancer Cells

**Figure 5:**
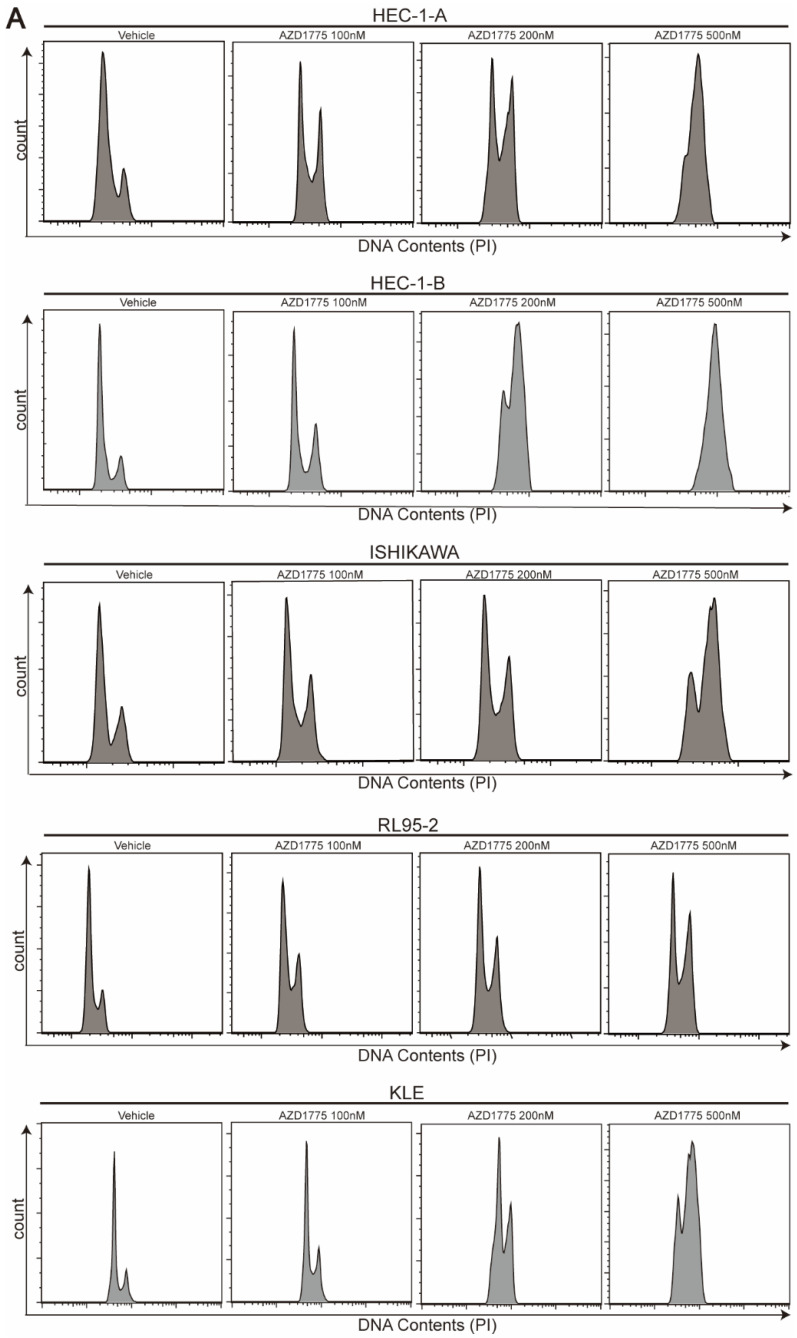
Influence of AZD1775 on Cell Cycle Progression in Endometrial Cancer Cells. A: Endometrial cancer cell lines HEC-1-A, HEC-1-B, ISHIKAWA, RL95-2, and KLE were seeded in 6-well plates and subjected to treatments with DMSO, 100 nM, 200 nM, and 500 nM of AZD1775 for 72 h. Post treatment, cell cycle distribution was analyzed using flow cytometry.

As a critical cell cycle regulator, WEE1 controls the G2/M checkpoint. Inhibition of WEE1 negates this checkpoint function, precipitating mitotic catastrophe. Previous research suggests that WEE1 inhibitors promote the premature entry of cancer cells into mitosis, but delay their exit, resulting in mitotic arrest and apoptosis^22^. To assess the effect of AZD1775 on cell cycle progression in endometrial cancer, we treated the five cell lines with varying concentrations of AZD1775 for 72 h. Consistent with prior studies, we found that AZD1775 induced G2/M cell cycle arrest in all five endometrial cancer cell lines (Figure 5). Remarkably, even a small dose of 100nM AZD1775 could significantly induce G2/M arrest in a concentration-dependent manner. These results confirm that AZD1775 significantly impacts cell mitosis and proliferation.

### WEE1 Inhibitors Activate the Innate Immune Response in Endometrial Cancer Cells

Previous studies have demonstrated that WEE1 inhibitors can lead to significant accumulation of dsDNA within the tumor cell cytoplasm^23^ which is subsequently recognized by cyclic GMP-AMP synthase. This recognition process triggers cyclic GMP-AMP synthase to signal cyclic guanoadenylic acid, thereby activating the stimulator of interferon (IFN) genes pathway. This cascade culminates in the phosphorylation of its downstream TANK binding kinase 1 (TBK1) and IFN regulator 3 (IRF3), ultimately inciting chemokine and IFN responses^24^.

To delineate the mechanisms underlying the impact of WEE1 inhibitors on endometrial cancer, we treated HEC-1-A, HEC-1-B, ISHIKAWA, RL95-2, and KLE cell lines with different concentrations of AZD1775 for 72 h. Following this, we performed western blotting to assess the expression levels of pertinent proteins.

**Figure 6:**
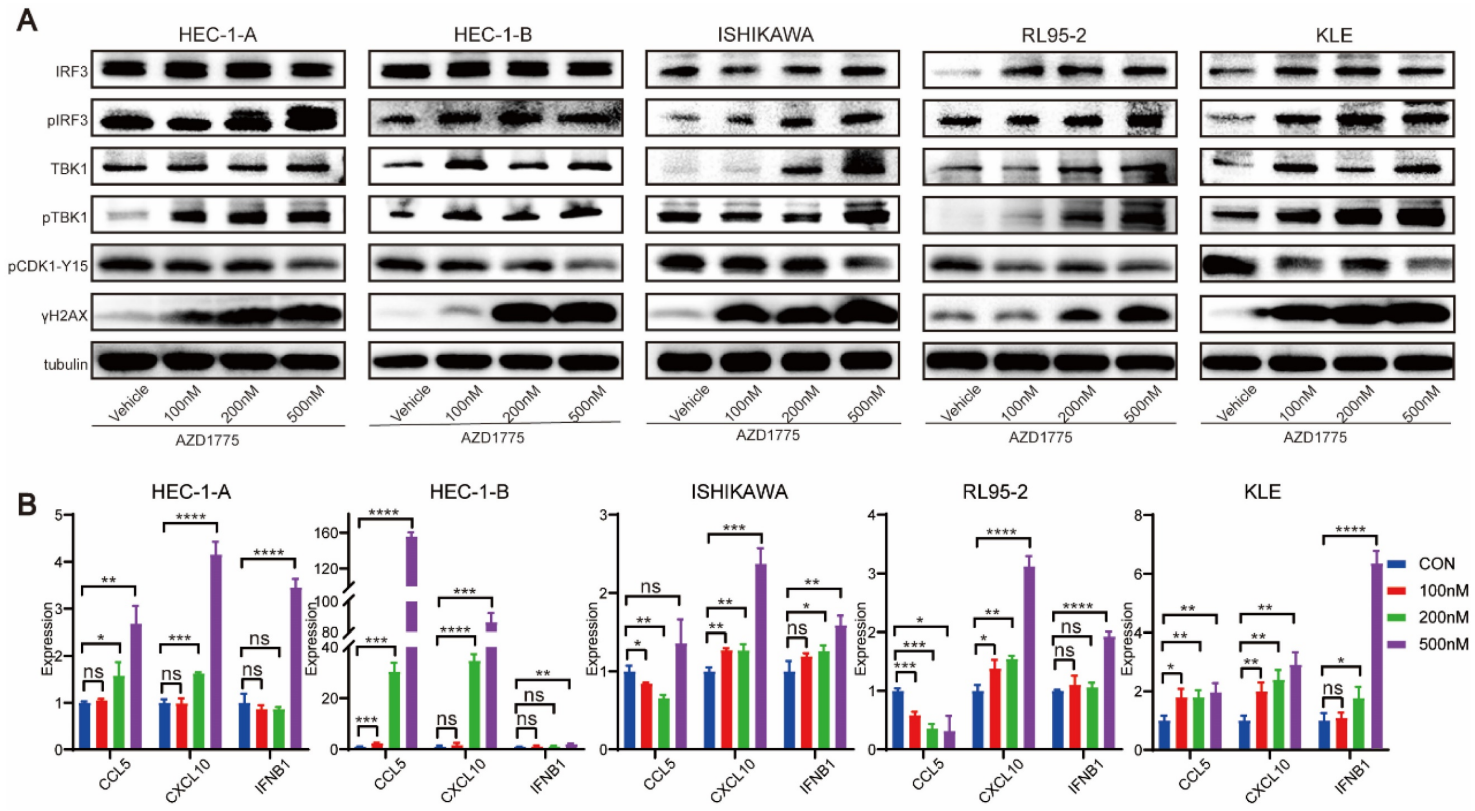
AZD1775 Induceed Innate Immune Activation in Endometrial Cancer Cells. A: Endometrial cancer cell lines HEC-1-A, HEC-1-B, ISHIKAWA, RL95-2, and KLE were subjected to treatments with DMSO, 100 nM, 200 nM, and 500 nM of AZD1775 for 72 hours. Subsequent to treatments, the expression of specific molecules was analyzed using Western Blotting assay. B: Upon similar treatment conditions, alterations in associated chemokines were evaluated using qRT-PCR assay. All experimental data were presented as mean ± standard deviation and were statistically assessed via t-test. Symbols denoted significance: * for P<0.05, ** for P<0.01, *** for P<0.001, **** for P<0.0001, and ns for non-significant.

Figure 6A demonstrates that as the drug concentration increased, WEE1 inhibitors elevated the expression level of γ-H2AX across the cell lines. γ-H2AX, a definitive marker of DNA double-strand breaks, signifies increased DNA damage when expressed highly. Furthermore, by phosphorylating the Tyr15 site of CDK1, WEE1 kinase facilitates the inspection and repair of damaged DNA, forestalling the entrance of abnormal DNA into mitosis, thereby preventing genomic instability. Western blotting analysis revealed that AZD1775 treatment diminished the expression level of pCDK1-Y15 in tumor cells (Figure 6A), a direct target of WEE1 kinase. These findings underscore the impact of AZD1775 on WEE1 kinase and its checkpoint role.

The subsequent experiment aimed to ascertain whether AZD1775 could activate the innate immune response pathway in endometrial cancer, with pTBK1 and pIRF3 serving as key effectors. Western blotting assay found that in five endometrial cancer cell lines, compared to the control group, AZD1775 treatment did not significantly alter the expression of total TBK1 and IRF3, but notably upregulated the expression of pTBK1 and pIRF3 (Figure 6A).

Previous data suggest that once pIRF3 enters the nucleus, it activates the type I IFN response, consequently stimulating the secretion of chemokines, Chemokine (C-C motif) ligand 5 (CCL5), and C-X-C Motif Chemokine Ligand 1 (CXCL10) in tumor cells^25^. Therefore, five endometrial cancer cell lines were treated with DMSO, 100nM, 200nM, and 500nM of AZD1775, and qRT-PCR was employed to measure the expression of CCL5, CXCL10, and IFNB1. Figure 6B indicates that as the drug concentration escalated, the expression of CCL5, CXCL10, and IFNB1 followed a similar rising trend.

Collectively, these results suggest that in endometrial cancer, AZD1775 significantly inhibits the checkpoint activity of WEE1 kinase, promotes DNA damage, and activates the innate immune signaling pathway.

### WEE1 Inhibitors Significantly Inhibit the Growth of Endometrial Cancer *In Vivo*

To further investigate the antitumor effects of AZD1775 on endometrial cancer and augment the clinical relevance of this study, we established *in vivo* tumor models using two endometrial cancer cell lines, HEC-1-B, and ISHIKAWA. Mice were randomly divided into two groups; one received solvent control gavage, and the other AZD1775 gavage. Body weight and tumor volume were measured every 3 days, with the mice sacrificed after 28 days. The body weight and tumor volume changes between the two groups were subsequently compared.

**Figure 7:**
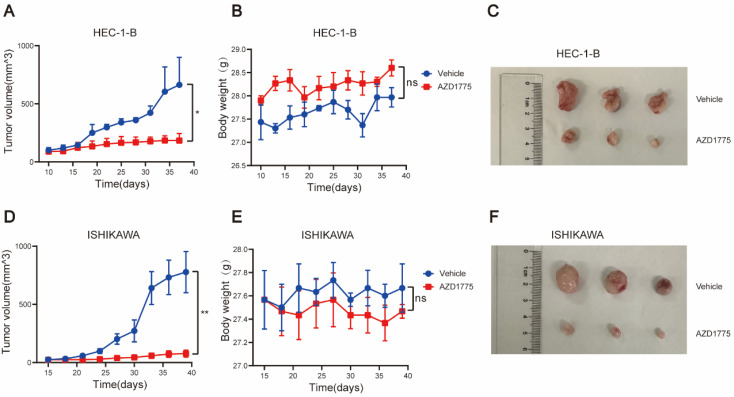
AZD1775 Demonstrated *In Vivo* Antitumor Activity in Endometrial Cancer. Endometrial cancer cell lines HEC-1-B and ISHIKAWA were subcutaneously inoculated into NCG mice. Upon tumors attaining a size of 2mm, mice were segregated into two cohorts: one received the solvent control (comprising 2% DMSO, 30% PEG300, 5% TWEEN80, and sterile PBS), and the other was administered AZD1775 at a dosage of 60 mg/kg/d, given once daily for 5 consecutive days each week, followed by a 2-day pause, over a 28-day period. A & D: Tumor volume progression curves in HEC-1-B and ISHIKAWA mouse models, determined using the formula: (length × width^2) × 3.14/6. B & E: Body weight variation curves for mice, across both treatment groups, within the HEC-1-B and ISHIKAWA models. C & F: Post-treatment subcutaneous tumor dimensions in HEC-1-B and ISHIKAWA mice. All experimental data were presented as mean ± standard deviation and were assessed using the t-test. Significance denoted as: * for P<0.05, ** for P<0.01, and ns for non-significance.

Our findings indicated that AZD1775 significantly inhibited tumor growth in HEC-1-B and ISHIKAWA mice post-tumorigenesis (Figure 7A, 7C, 7D, 7F). Furthermore, we monitored the mice's overall health status, including body weight. The results revealed no significant difference in body weight between the experimental and control groups (Figure 7B, 7E), suggesting that AZD1775's inhibitory effect on endometrial cancer did not compromise the overall health status of the mice.

## Discussion

Recurrent endometrial cancer poses significant therapeutic challenges due to limited treatment options. Surgical resection or radiotherapy can be pursued for patients with localized recurrence, whereas individuals with more extensive relapse typically receive multi-modal treatments, which often include surgery, radiotherapy, chemotherapy, and endocrine therapy^6^. Recently, targeted therapy has emerged as a promising strategy for recurrent endometrial cancer. DNA damage repair (DDR) molecules, integral to tumor progression, recognize and initiate repair pathways in response to substantial DNA damage^26^. Notably, cell cycle checkpoints, especially WEE1 kinase, play a crucial role in DNA repair processes^13^.

WEE1 kinase, adept at identifying damaged DNA, induces cell cycle arrest at the G2/M phase by phosphorylating the Tyr15 site of CDK1. This enables DNA repair and subsequent re-entry into the mitosis phase^27^. As endometrial cancer progression involves substantial DNA damage, the role of WEE1 kinase in maintaining normal mitosis becomes paramount. Accordingly, in this study, we used paraffin-embedded tumor tissue sections from endometrial cancer patients for immunohistochemical staining. Our findings showed high WEE1 kinase expression in endometrial cancer tissue, with significant higher expression level of WEE1 protein in recurrent patients (P<0.0001). Studies have indicated that WEE1 protein, a nuclear-expressed kinase regulating cell division progression, is associated with poor prognosis in various malignancies, such as malignant melanoma^28^, glioblastoma^29^ and ovarian cancer^21^. Consistently, our study revealed that overexpression of WEE1 protein was statistically linked with unfavorable prognosis in endometrial cancer patients.

AZD1775 represents the sole WEE1 kinase inhibitor employed in fundamental studies and clinical trials, due to its capacity to bind to WEE1 protein kinase and negate its checkpoint function at G2/M. Previous research identified AZD1775 as a promising targeted therapy, capable of augmenting the efficacy of both radiotherapy and chemotherapy, thereby improving patient survival^30^, ^31^. Besides solid tumors, targeting WEE1 kinase has shown promising therapeutic activity in hematological malignancies and is anticipated to enhance the effectiveness of several drugs^32^. However, with respect to endometrial cancer, extensive exploration is necessary to fully elucidate the therapeutic potential of targeting WEE1 kinase. In the present study, we selected and cultured five endometrial cancer cell lines: HEC-1-A, HEC-1-B, ISHIKAWA, RL95-2, and KLE. Initially, we utilized a CCK8 assay to determine the cytotoxic effect of AZD1775 on endometrial cancer cells and monitored cell survival after 72 hours of treatment. AZD1775 exerted a potent inhibitory effect on all five cell lines relative to the control group. Additionally, we conducted clone proliferation assays, wound healing assays, and transwell assays to evaluate the impact of AZD1775 on endometrial cancer cell proliferation and migration. Our results indicate that AZD1775 effectively inhibits tumor cell proliferation and migration, thereby impeding endometrial cancer progression.

Besides, five endometrial cancer cell lines were treated with different concentrations of AZD1775 and flow cytometry was performed to detect cell apoptosis 72 h later. Though AZD1775 at a concentration of 100nM didn't cause tumor cell death, it induced apoptosis in a concentration-dependent manner from 200nM. RL95-2 didn't show an increase in cell death after 72 h of drug treatment, possibly because of its relative slow proliferation and insensitivity to the low concentration of drug administration. However, consistent with the other four cell lines, RL95-2 demonstrated significant apoptosis from a drug concentration of 500nM. Given reports that protein expression level changes are more sensitive post-dosing, we performed western blotting to detect the expression of apoptotic protein, caspase 3. Even at an AZD1775 concentration of just 100nM, caspase 3 displayed an upward trend in all endometrial cancer cell lines, suggesting that AZD1775 can induce tumor cell apoptosis to exert antitumor effects, even at relatively low concentrations. Additionally, AZD1775 was shown to induce cell cycle arrest, with flow cytometry analyses indicating G2/M phase arrest in endometrial cancer cells from a concentration of 100nM, in a dose-dependent manner. These *in vitro* experiments confirmed that AZD1775 has significant inhibitory effects on endometrial cells, influencing cell division and proliferation. A study by Dasari et al.^33^ combined an EphA2 targeted agent with AZD1775 in HEC-1-A and ISHIKAWA cell lines and found that combination therapies reduced cell viability and clone formation ability while inducing cell death. These findings align with the effects observed with AZD1775 monotherapy in our study. Notably, the reported study utilized a dose of 500nM AZD1775 in the combination therapy, whereas our study demonstrated that AZD1775, used as monotherapy at a lower dose of 100nM, could still yield significant antitumor activity.

Furthermore, endometrial cancer mouse models were established using HEC-1-B and ISHIKAWA cell lines, and these were administered 60mg/kg/day of AZD1775. Regular assessments of tumor volume and mouse body weight were conducted, and comparisons between groups were made following 28 days of drug administration. We observed that, relative to the control group, AZD1775 substantially inhibited tumor growth without impacting mouse development, and the tumor suppression rate reached 70%.

Targeted therapies typically result in augmented DNA damage. WEE1 inhibitors may disrupt the DNA repair process, leading to an accumulation of unrepaired damaged DNA within the cytoplasm. The cyclic GMP-AMP synthase pathway recognizes this damaged DNA in the cytoplasm, triggering cyclic guanoadenylic acid signaling and subsequently activating the stimulator of IFN genes -related innate immune response pathway. This process results in the phosphorylation of downstream TBK1 and IRF3, further stimulating chemokines and IFN responses^34^. In this investigation, endometrial cancer cells were treated with 100nM, 200nM, and 500nM AZD1775, and western blotting was employed to detect alterations in associated protein expressions. We found that AZD1775, compared with the control group, reduced the expression levels of pCDK1 Y15 (the direct target of the WEE1 protein) in a concentration-dependent manner. This facilitated increased DNA damage in endometrial cancer and activation of the downstream innate immune response pathway. While the overall levels of TBK1 and IRF3 in HEC-1-A and HEC-1-B endometrial cancer cell lines did not change significantly, the expression levels of pTBK1 and pIRF3 increased. In the other three cell lines, both overall and phosphorylated TBK1 and IRF3 significantly increased. Given the effector roles of phosphorylated TBK1 and IRF3, AZD1775 was shown to activate the innate immune response signaling pathway in endometrial cancer cell lines.

Previous reports suggested that the administration of targeted therapies can lead to the activation of pTBK1 and pIRF3, thus instigating the release of chemokines and IFN and subsequently stimulating downstream inflammatory responses^34^. Accordingly, we treated five endometrial cancer cell lines with varying concentrations of AZD1775 for 72 hours and conducted a qRT-PCR assay to assess the expression of chemokines in the tumor cells. The findings indicated an upregulation in the expression of CCL5 and CXCL10 across all cell lines following AZD1775 treatment. Furthermore, we observed an induction in the release of IFNB1, albeit insignificant at lower concentrations of AZD1775 across the five cell lines. In a study on non-small cell lung cancer, TANIGUCHI et al.^25^ demonstrated that AZD1775 could activate the stimulator of IFN genes -TBK1-IRF3 pathway, stimulating the release of pro-inflammatory chemokines CCL5 and CXCL10, as well as type I IFN. These results are in line with our findings, indicating that WEE1 inhibitors may exert antitumor effects by triggering innate immune response pathways in endometrial cancer cells.

Our study revealed that the expression levels of the WEE1 protein were significantly higher in tumor tissues of patients with recurrent endometrial cancer, and were associated with clinical prognosis. A series of *in vitro* and *in vivo* experiments confirmed the potent antitumor effects of WEE1 inhibitors on endometrial cancer, which appeared to be related to the activation of the innate immune response. Nevertheless, our study has certain limitations. Currently, targeted therapy is primarily employed in combination with other treatments, effectively enhancing antitumor effects and reducing drug resistance. However, our study did not thoroughly investigate the signaling pathway through which WEE1 inhibitors exert their antitumor effects in endometrial cancer or explore potential combination therapy strategies. Additionally, while research indicates that WEE1 inhibitors show synthetic lethality in P53 mutant tumors^35^, and TP53 mutation is associated with a worse prognosis in endometrial cancer, we observed that AZD1775 exhibited strong cytotoxic effects in P53 mutant endometrial cancer. We did not further investigate the relationship between TP53 mutation and the antitumor efficacy of WEE1 inhibitors. Future work will refine these areas, identifying the patient population most responsive to WEE1 inhibitor treatment to facilitate the development of precision adjuvant treatment options.

## Conclusion

In this study, *in vitro* experiments demonstrated WEE1 protein is expressed in the cancer tissues of endometrial cancer patients. Compared with non-recurrent patients, WEE1 protein is significantly overexpressed in the cancerous tissues from patients with recurrent endometrial cancer. Crucially, WEE1 inhibitors demonstrated potent antitumor effects by not only inducing DNA damage and disrupting DNA repair, but also activating innate immune response pathways and promoting the release of chemokines and IFNs. These combined actions effectively suppressed cellular proliferation and promoted apoptosis. Concurrently, *in vivo* analyses revealed that WEE1 inhibitors robustly hampered tumor occurrence and progression, without detrimental impacts on the growth parameters of the host mice, such as body weight. This research underscores the significant potential of WEE1 inhibitors in the therapeutic arsenal against endometrial cancer, particularly for patients with recurrent disease and poor prognosis. Thus, WEE1 inhibitors are anticipated to offer a promising novel avenue to extend survival times for patients suffering from endometrial cancer.

## Supplementary Material

Supplementary tables.
