# Supplementary material for: WEE1 Inhibitors Mediate Antitumor Effects on Endometrial Cancer through Activation of Innate Immune Responses
Source: J Cancer. 2024 Jan 1;15(2):545–59. doi: 10.7150/jca.90236 (PMC10758029; doi:10.7150/jca.90236)
Supplement: Supplementary file 1 — Supplementary tables. [file jcav15p0545s1.pdf]

Supplementary Information Table S1. List of Antibodies

Antibodies	Source	Identifier
β-tubulin	ABclonal	A12289
γH2AX	CST	9718S
Caspase 3	Abcam	Ab184787
pCDK1-Y15	ABclonal	AP0016
TBK1 A3458	ABclonal	A3458
IRF3	SinoBiological	108076-732
Phospho-TBK1	ABclonal	AP1026
Phospho-IRF3	ABclonal	AP0995
WEE1	Abcam	Ab288727

Supplementary Information Table S2. List of Primers

**Gene name**	**Forward primer**	**Reverse primer**
β-actin	CATGTACGTTGCTATCCAGGC	CTCCTTAATGTCACGCACGAT
CCL5	TGCTGCTTTGCCTACATTGC	CATCCTTGACCTGTGGACGA
CXCL10	CCACGTGTTGAGATCATTGCT	TGCATCGATTTTGCTCCCCT
IFNB1	AGTAGGCGACACTGTTCGTG	GCCTCCCATTCAATTGCCAC
